# The Role of Bioelectrical Impedance Analysis in Predicting Secondary Surgical Interventions for Lymphedema

**DOI:** 10.3390/jcm14072151

**Published:** 2025-03-21

**Authors:** Wataru Otsuka, Shuhei Yoshida, Nanami Taketomi, Yasushi Orihashi, Isao Koshima

**Affiliations:** 1 Plastic Surgery & International Center for Lymphedema (ICL), Hiroshima University Hospital, 1-2-3, Kasumi, Minami-ku, Hiroshima 734-8551, Japan; syuheiyoshida44@gmail.com (S.Y.); koushimaipla@gmail.com (I.K.); 2 Clinical Research Center in Hiroshima, Hiroshima University Hospital, 1-2-3, Kasumi, Minami-ku, Hiroshima 734-8551, Japan; nnmtk73@hiroshima-u.ac.jp (N.T.); y-orihashi@hiroshima-u.ac.jp (Y.O.)

**Keywords:** lymphedema, bioelectrical impedance analysis, lymphaticovenular anastomosis, extracellular water

## Abstract

**Background**: Bioelectrical impedance analysis (BIA), known for its utility in monitoring fluid balance and lymphedema progression, is non-invasive and practical. However, circumferential tape measurements remain the gold standard for assessing limb volume changes, despite operator variability. This study investigated whether BIA could reliably assess the need for secondary surgical interventions in lymphedema patients. **Methods**: We retrospectively analyzed lower extremity lymphedema patients who underwent multiple lymphaticovenous anastomoses on both legs from April 2017 to June 2023. This study involved 14 patients with a single surgery and 34 requiring additional surgeries. Logistic regression evaluated associations between the number of surgeries and valuables, including extracellular water-to-total body water (ECW/TBW) ratios measured via BIA, the sum of five-part circumferential values via tape measuring, age, and body mass index. Receiver operating characteristic (ROC) curve analysis calculated the area under the curve (AUC) for ECW/TBW and circumference values, analyzed separately for left and right legs. **Results**: ECW/TBW values were significantly associated with the need for a second surgery for both the right leg (*p* = 0.02, ROC-AUC = 0.86) and the left leg (*p* = 0.04, ROC-AUC = 0.86). In contrast, circumference measurements were not significant predictors for either the right leg (*p* = 0.46, ROC-AUC = 0.77) or the left leg (*p* = 0.60, ROC-AUC = 0.78). ECW/TBW demonstrated a higher AUC compared to circumference measurements, indicating its potential as a more sensitive tool for predicting the need for additional surgical interventions. **Conclusions**: BIA may serve as a valuable tool for monitoring treatment outcomes and guiding secondary surgical planning. Larger studies are needed to validate its clinical utility.

## 1. Introduction

Lymphedema is a condition characterized by impaired lymphatic drainage, leading to the accumulation of lymph fluid in the interstitial compartment. Primary lymphedema results from structural abnormalities in the lymphatic system, where proximal lymph flow is reduced or stagnant despite the presence of collecting vessels [1]. Secondary lymphedema, which is far more common, arises from damage or obstruction of previously normal lymphatic pathways due to malignancies, infections, trauma, surgery, obesity, or radiation therapy [2]. Surgical interventions become necessary when conservative therapies fail to adequately control edematous changes [3].

Microsurgical lymphaticovenular anastomosis (LVA), first described by O’Brien et al. in 1977 [4,5], has since evolved with the advent of supermicrosurgical techniques introduced by Koshima, significantly improving the success rates and predictability of the procedure [6]. Surgical options for lymphedema primarily include LVA, vascularized lymph node transfer (VLNT), liposuction (LS), and others. Among these, LVA and VLNT have gained popularity for enhancing lymphatic drainage and reducing edema [7]. Various diagnostic methods for lymphedema also exist, each with its own advantages and limitations.

Bioelectrical impedance analysis (BIA), first applied clinically in 1992, measures extracellular water (ECW), including lymph fluid, and has advanced significantly in instrumentation and data analysis [8]. As a result of an accumulation of excess lymph fluid, lymphedema leads to an increase in the total amount of extracellular fluid in the affected limb [9]. BIA estimates the body’s impedance to electrical currents, which correlates inversely with fluid levels. It segments the body into five regions (right arm, left arm, trunk, right leg, and left leg) and estimates intracellular water (ICW), ECW, and total body water (TBW) in each compartment. The fat-free mass, skeletal muscle mass, and edema index (defined as the ECW/TBW ratio) are also calculated. The lymphedema index (L-Dex) is a quantitative measure used to assess lymphedema by comparing limb impedance ratios [10,11]. Some BIA devices automatically calculate the L-Dex, simplifying lymphedema assessment and making it easier to interpret across different populations. BIA has a high intra- and inter-rater reliability, and previous studies have demonstrated the utility of BIA in monitoring lymphedema progression and evaluating the efficacy of lymphedema management strategies, including surgical interventions [12,13,14,15]. Nevertheless, circumferential tape measurements remain the gold standard for assessing limb volume changes, despite being prone to operator variability and lacking comprehensive fluid distribution assessment [16].

Moreover, the treatment of advanced lymphedema is not straightforward. Ideally, lymphedema could be resolved with a single, but in advanced cases, LVA alone is often insufficient due to lymphatic sclerosis [17]. For patients with advanced lymphedema, a second surgery, such as additional LVA, VLNT, or LS, is often required because the lymphatic drainage function cannot be fully restored with initial LVA alone [3,18]. However, determining the optimal timing for a second surgical intervention remains a clinical challenge [19]. To the best of our knowledge, no high-quality evidence exists regarding standardized methods to guide secondary treatment decisions for lymphedema.

This study aimed to evaluate the outcomes of LVA using BIA and investigate the potential of this technology in guiding decisions for additional surgeries. Through comparative analysis, we assessed whether BIA and circumferential tape measurements could reliably inform surgical decision-making.

## 2. Materials and Methods

### 2.1. Patients and Study Design

A retrospective cohort study was conducted on patients who underwent multiple LVAs for lymphedema at a single hospital. Written informed consent was obtained from all participants.

Inclusion criteria were as follows:

1. Age between 18 and 65 years.
2. A confirmed diagnosis of LEL based on medical history, physical examination, BIA, and indocyanine green (ICG) lymphography.
3. Edema refractory to 6 months of supervised conventional compression therapy using a pressure garment
4. No prior surgical treatment for LEL.
5. Initial multiple LVAs performed on both legs.
6. At least one preoperative and two postoperative measurements of limb circumference and BIA, performed on the same day.
7. Regular follow-ups preoperatively (at 3 and 6 months).
8. A minimum of one year has elapsed since the initial LVA.

Exclusion criteria were as follows:

1. Unilateral LVA procedures.
2. Severe cases requiring primary VLNT or combined surgeries.
3. Cases where the interval between surgeries was insufficient to allow for two postoperative measurements.

### 2.2. Tape Measuring

Circumference measurements of the lower limbs were taken at five anatomic locations: 10 cm above the knee, at the knee, 10 cm below the knee, the ankle, and the foot. Measurements were performed by a trained nurse or physiotherapist who was uninvolved in this study in order to minimize bias. All measuring staff were well-trained in standardized measurement techniques and received periodic training. Patients were randomly assigned to the measuring staff during outpatient visits.

### 2.3. Segmental Multifrequency BIA

Segmental multifrequency (SMF)-BIA was conducted using a portable InBody S10 device (InBody, Tokyo, Japan). The device employs six frequencies (1, 5, 50, 250, 500, and 1000 kHz) to analyze segmental body composition and fluid status. A weak alternating current is applied to four specific points (typically the wrists and ankles), automatically calculating and recording intracellular water (ICW), ECW, TBW, and the ECW/TBW ratio. BIA was performed once for each patient on the same day as the tape measurements, with the same staff member conducting both procedures.

### 2.4. LVA

LVA was performed precisely along a line traced by making the superficial lymphatic vessels if ICG lymphography indicated a linear pattern and along the course of the greater saphenous vein if ICG lymphography showed a dermal backflow pattern or no enhancement. Multiple LVA procedures were performed on both legs under local anesthesia by surgeons proficient in supermicrosurgery. Using microscopic magnification, several 1- to 5-cenitimeter skin incisions were made in the lower leg and thigh regions, and lymphatic vessels were anastomosed to adjacent veins in either an end-to-end or end-to-side fashion.

### 2.5. Data Collection

Data were collected from patients who underwent multiple BIA and circumference measurements between April 2018 and June 2023. The following variables were recorded: ECW/TBW ratio, sum of five circumference measurements, age, BMI, and number of surgeries (single vs. multiple procedures). The severity of lymphedema was categorized using the International Society of Lymphology (ISL) classification. The etiology of lymphedema was categorized as primary or secondary, and in the case of secondary lymphedema, the underlying cause was also classified.

### 2.6. Statistical Analyses

Data analysis focused on the ECW/TBW ratio from the BIA and the sum of the five tape-measured values, evaluated separately for the left and right legs. Patients were divided into the following two groups:

1. Patients who underwent a single surgery with no subsequent procedures.
2. Patients who required additional surgeries.

To investigate whether BIA or tape measuring could assess the need for secondary surgical interventions, the most recent BIA and circumference data from the single-surgery group were compared with the data collected just prior to additional surgeries from the second group.

Nominal logistic regression analysis was performed to evaluate the association between the number of surgeries (one or two) and ECW/TBW, the sum of the five tape-measured values of tape measuring, age, and BMI. The number of surgeries was set as the dependent variable, while ECW/TBW, total circumference values, age, and BMI were included as explanatory variables in the model. Receiver operating characteristic (ROC) curve analysis was calculated to determine the area under the curve (AUC) for ECW/TBW and total circumference values. The fit of two logistic regression models was compared to determine whether ECW/TBW or circumferential sum was a better predictor of the need for a second surgery. Model performance was assessed using the ROC-AUC. Analyses were conducted separately for the left and right legs, and a value of *p* < 0.05 was considered to be statistically significant. Statistical analyses were performed using JMP Pro, version 18.1.1 (SAS Institute Inc., Cary, NC, USA).

## 3. Results

### 3.1. Patient Characteristics

In total, 320 patients underwent multiple LVAs on both legs. After excluding ineligible cases, 48 patients were included in this study (Table 1). Fourteen (29.2%) patients underwent a single surgery without additional procedures, while 34 (70.8%) required additional surgeries. There were 8 males (16.7%) and 40 females (83.3%). The mean age was 53.2 years (range 32–65; SD 9.7). The mean BMI was 24.7 (range 16.9–53.5; SD 6.6). The etiologies of lymphedema were as follows: primary (20 cases), uterine cervical cancer (14 cases), uterine corpus cancer (9 cases), ovarian cancer (1 case), and other malignancy (4 cases). Clinical staging based on the ISL classification was as follows: stage I (12 patients), stage II (29 patients), late class II (6 patients), and class III (1 patient). All surgical procedures were conducted without complications, such as lymphorrhea or delayed wound healing. No patient developed cellulitis during the study period.

### 3.2. Logistic Regression and ROC-AUC for Predicting the Need for a Second Surgery

Four separate logistic regression analyses were conducted to evaluate the association of preoperative metrics with the need for a second surgery.

**Figure 1:**
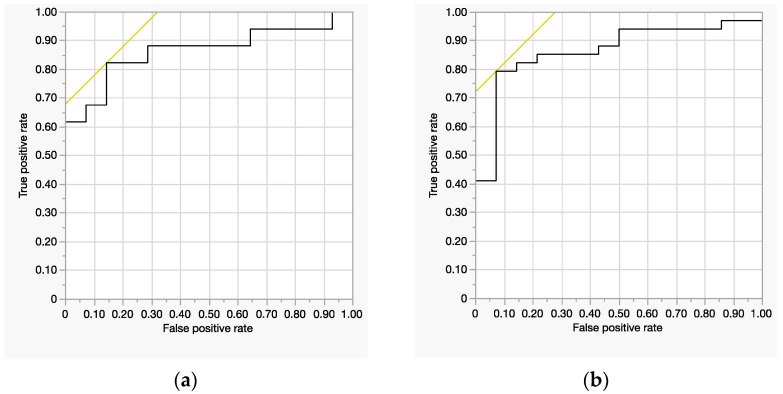
Receiver operating characteristic (ROC) curve: (**a**) Right leg ECW/TBW. (**b**) Right leg circumferential sum.

**Figure 2:**
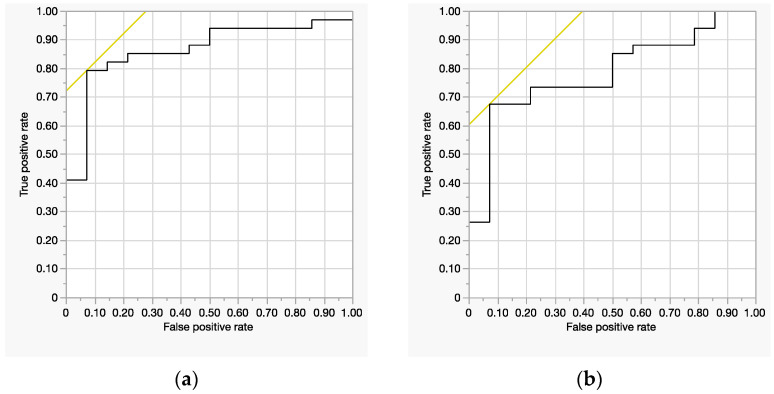
Receiver operating characteristic (ROC) curve: (**a**) Left leg ECW/TBW. (**b**) Left leg circumferential sum.

1. Right leg ECW/TBW (BIA).
  - Model Fit: Significant (*χ*^2^ = 17, *p* = 0.0006, *R*^2^ = 0.30).
  - Significant Predictors: ECW/TBW (*p* = 0.021), age (*p* = 0.0025), and BMI (*p* = 0.043).
  - ROC-AUC: 0.86 (good discrimination) (Figure 1a).
2. Right leg circumferential sum (tape measuring).
  - Model Fit: Significant (*χ*^2^ = 10, *p* = 0.015, *R*^2^ = 0.18).
  - Significant Predictor: age (*p* = 0.0093). The circumferential sum was not significant (*p* = 0.46).
  - ROC-AUC: 0.77 (acceptable discrimination) (Figure 1b).

1. Left leg ECW/TBW (BIA).
  - Model Fit: Significant (*χ*^2^ = 17, *p* = 0.0007, *R*^2^ = 0.29).
  - Significant Predictors: ECW/TBW (*p* = 0.037), age (*p* = 0.0045), and BMI (*p* = 0.042).
  - ROC-AUC: 0.86 (good discrimination) (Figure 2a).
2. Left leg circumferential sum (tape measuring).
  - Model Fit: Significant (*χ*^2^ = 10, *p* = 0.017, *R*^2^ = 0.18).
  - Significant Predictor: age (*p* = 0.013). The circumferential sum was not significant (*p* = 0.60).
  - ROC-AUC: 0.78 (good discrimination) (Figure 2b).

Logistic regression models revealed that ECW/TBW was significantly associated with the need for a second surgery, whereas circumferential sum showed no significant association (Table 2).

### 3.3. ROC-AUC Comparisons

- Right leg AUC difference: 0.090 (*p* = 0.10)
- Left leg AUC difference: 0.084 (*p* = 0.051)
- The ROC-AUC values of models incorporating ECW/TBW were consistently higher than those using circumferential sum, though statistical significance was not achieved (Table 3).

## 4. Discussion

Circumferential tape measurements, a commonly used method for assessing lymphedema, have been reported to correlate with BIA [20,21]. Tape measurements are considered to have high inter-rater reliability when performed by a well-trained physiotherapist, whereas they are also reported to be prone to variability, potentially leading to false-negative or false-positive results [22,23]. Advanced imaging modalities, such as lymphoscintigraphy and ICG lymphography, have also been explored to evaluate the efficacy of LVA [24]. While these methods provide detailed and valuable insights, their high cost and limited accessibility make them less practical for routine clinical use. In contrast, BIA offers a non-invasive, simple, and rapid assessment tool [25]. Its relative affordability and ease of use make it well accepted by patients and suitable for repeated measurements in various clinical settings. Previous studies have highlighted its utility in monitoring fluid balance and evaluating lymphedema progression [26,27]. However, no high-quality evidence exists regarding methods to guide secondary treatment decisions for severe lymphedema.

The statistical analyses revealed that for ECW/TBW, the right side showed a significant predictor (*p* = 0.021), and the left side also showed a significant predictor (*p* = 0.037). On the other hand, the circumference measurements showed no significant predictors for either the right side (*p* = 0.46) or the left side (*p* = 0.60). Thus, our study demonstrated that ECW/TBW values were significantly associated with the need for a second surgery. In contrast, circumference measurements showed no significant association with it in the individual analysis. Importantly, ECW/TBW values showed consistently higher AUC values compared to circumference measurements, though statistical significance was not achieved.

The trend of higher AUC values for ECW/TBW suggests that BIA may provide a more sensitive and objective metric for postoperative evaluation compared to circumference measurements. This aligns with prior studies emphasizing the sensitivity of BIA in monitoring fluid balance in lymphedema patients [12,28]. Furthermore, BIA might be able to predict the outcomes of lymphedema treatment [29] and more sensitively reflect the effectiveness of LVA, making it a valuable tool for tracking disease progression and evaluating the efficacy of surgical interventions [25,26,30]. In addition to its established role in the early diagnosis, treatment evaluation, and outcome prediction of lymphedema, as demonstrated in various previous studies, our findings suggest that BIA may serve as a useful method for planning subsequent treatment procedures. The ability of BIA to detect subtle changes in extracellular fluid accumulation suggests that it could serve as an objective tool for determining the optimal timing of secondary surgical interventions. Unlike traditional circumferential tape measurements that rely on subjective clinical examination, BIA provides quantifiable data that can guide treatment decisions. By identifying early signs of worsening lymphatic function, BIA may help optimize the timing of additional procedures, thereby preventing unnecessary delays in intervention while avoiding premature surgical decisions. Furthermore, integrating BIA with other tools—such as ultrasound or ICG lymphography—may provide a more comprehensive assessment of lymphedema. Combining the quantitative fluid information from BIA with the structural and functional insights obtained through these examinations could enhance the accuracy of disease evaluation and improve treatment planning, ultimately leading to better patient outcomes.

In this study, age was also identified as a significant predictor across all analyses, suggesting that older patients are more likely to require additional interventions. This may be attributed to the decline in lymphatic function associated with aging [31]. Since the progression of lymphedema is associated with aging [32], there is a possibility that lymphedema may advance over time even if the initial surgery is successful. Some patients tend to discontinue compression therapy once their edema improves with surgery and avoid attending regular follow-up visits. Lymphedema may frequently recur due to the discontinuation of the compression therapy or aging. Since patient education is an important component of lymphedema prevention, it is essential to present easily understandable examination results and clearly explain the necessity of additional surgical interventions [33]. Long-term outpatient monitoring and follow-up using BIA for advanced lymphedema patients can be considered an efficient approach. BIA, which sensitively reflects the need for secondary surgical interventions during repeated long-term follow-up, is likely to play a central role in the future management of lymphedema.

Our study had several limitations. First, this study is limited by its small sample size and single-center cohort study challenges, which limited the generalizability of our findings. A well-designed study with a larger population is needed. Second, the diversity in patient backgrounds, including comorbidities like cellulitis, may have influenced the outcomes. Recurrent cellulitis can lead to progressive damage to the lymphatic system [34]. It means that there were differences in surgical resistance between the right and left limbs, which might have influenced the observed outcomes. An analysis of BIA according to the frequency of cellulitis episodes may also be needed. Third, there was also variability in ISL staging among patients, which may have contributed to differences in disease severity and treatment responses. Future studies with larger cohorts are required to validate the predictive superiority of ECW/TBW measurements.

Despite these limitations, our findings provide compelling preliminary evidence that the ECW/TBW ratio derived from BIA is a promising tool for predicting the need for secondary surgical interventions. This suggests that BIA may be integrated into routine clinical practice for more accurate and timely monitoring of lymphedema. Further prospective, multicenter studies are warranted to confirm these findings and to refine the role of BIA in the management of lymphedema.

## 5. Conclusions

While ECW/TBW demonstrated promising trends in predictive accuracy over circumference measuring, further validation through larger studies is essential. BIA holds potential as a superior metric for predicting secondary surgical interventions in lymphedema management.

## Figures and Tables

**Table 1:** Patient characteristics.

Characteristics		Value
Number of patients		48
Number of surgeries	Single (%)	14 (29.2)
	Multiple (additional) (%)	34 (70.8)
Sex	Male (%)	8 (16.6)
	Female (%)	40 (83.3)
Age (years)	Average (range)	53.2 (32–65)
BMI	Average (range)	24.7 (16.9–53.5)
ISL stage	I (%)	12 (25)
	II (%)	30 (62.5)
	Late II (%)	6 (12.5)
	III (%)	1 (2.0)
Etiology of lymphedema	Primary (%)	20 (41.7)
	Uterine cervical cancer (%)	14 (29.2)
	Uterine corpus cancer (%)	9 (18.8)
	Ovarian cancer (%)	1 (2.0)
	Other malignancy (%)	4 (8.3)

BMI: body mass index; ISL: International Society of Lymphology classification.

**Table 2:** Logistic regression results for predicting the need for a second surgery.

	Predictor	Estimates	*p*
Right leg ECW/TBW (BIA)	ECW/TBW	96	0.021
	age	−0.21	0.0025
	BMI	−0.12	0.043
Right leg circumferential sum (tape measuring)	Circumferential sum	0.02	0.46
	age	−0.12	0.0093
	BMI	−0.12	0.15
Left leg ECW/TBW (BIA)	ECW/TBW	97	0.037
	age	−0.17	0.0003
	BMI	−0.13	0.034
Left leg circumferential sum (tape measuring)	Circumferential sum	0.017	0.60
	age	−0.11	0.013
	BMI	−0.11	0.22

ECW/TBW: extracellular water-to-total body water ratio; BMI: body mass index.

**Table 3:** Comparison of AUC values for predicting the need for a second surgery.

Predictor	AUC	95% CI	AUC Diff.	SE (Diff.)	*p*
Right leg ECW/TBW	0.86	0.72–0.94			
Right leg circumference sum	0.77	0.60–0.88	0.090	0.055	0.10
Left leg ECW/TW	0.86	0.71–0.94			
Left leg circumference sum	0.78	0.61–0.88	0.084	0.043	0.051

ECW/TBW: extracellular water-to-total body water ratio; AUC: area under the curve; CI: confidence interval; Diff: difference; SE: standard error.

## Data Availability

The data presented in this study are available upon request from the corresponding author.
